# Mild cognitive impairment: not much harm; not much help

**DOI:** 10.1192/bjo.2023.565

**Published:** 2023-10-10

**Authors:** Jeremy D. Isaacs

**Affiliations:** St George's, University of London, UK

**Keywords:** Dementias and neurodegenerative diseases, patients, mild cognitive impairment, memory services, biomarkers

## Abstract

Mild cognitive impairment (MCI) represents a liminal state between full cognitive health and dementia. The diagnosis is applied unevenly and cannot be accurately prognosticated, even with the use of biomarkers, and there is no established intervention to reduce risk of progression to dementia. Owing to the limited benefit and potential for iatrogenic harm associated with an MCI diagnosis, a better understanding of its psychosocial consequences is needed. In the linked paper, Munawar and colleagues provide cautious optimism; their patients were generally unharmed by an MCI diagnosis. However, the majority of patients and families either did not recall or did not fully understand the implications for future dementia risk. Only 20% made lifestyle changes, and the number receiving hearing aids was very low. These data demonstrate the poor return on using the clinic as the setting for improving ‘brain health’. Initiatives to prevent dementia are more effectively and equitably applied at population level.

Despite refinements in dementia clinicopathological correlations in the 1990s and 2000s, tools to confirm brain pathology during life remained limited. Reflecting this, clinical diagnostic criteria for dementia were conservative, requiring impairment in both cognitive and social function. With increasing public and professional awareness of Alzheimer's disease and other dementias, clinicians in the burgeoning memory clinic industry were faced with patients presenting with cognitive complaints typical of those with dementia, who performed below expectations on neuropsychometric tests but did not display impairment in activities of daily living. The concept of mild cognitive impairment (MCI) evolved to facilitate clinical management of and research into this liminal state between complete cognitive health and dementia.^1^

What to tell such patients? Send them away with reassurance that they don't have dementia? Or inform them that they have a pre-dementia state and can expect to progress to dementia within a short space of time? The difficulty facing clinicians is that either of these predictions could be accurate in any particular MCI patient. About 50% of MCI patients have not progressed to dementia after 5 years, and a significant minority revert, at least temporarily, to normal cognitive function.^2^

MCI is itself a highly unstable construct, used with extreme variability between clinicians and clinical settings. In the 2019 English national memory service audit, the frequency with which it was diagnosed varied among participating services from 0 to 47% of patients.^3^ Variation exists in defining both whether cognitive underperformance reaches threshold, with many MCI diagnoses given without formal neuropsychological assessment, and whether activities of daily living remain unimpaired, a judgement that is highly contextual and influenced by social and cultural factors. Amnestic MCI is more likely to progress to Alzheimer's dementia than non-amnestic MCI, but further reproducible clinical indicators of progression from MCI to dementia such as multiple-domain cognitive involvement have proved elusive.^4^ An alternative approach, albeit still in development, has been to use conversation analysis to categorise differences in how patients with dementia and those with functional cognitive disorder, an important and still under-recognised diagnosis, describe their symptoms.^5,6^

The advent of dementia biomarkers has been hailed by some as a route out of this uncertainty. These are best developed in Alzheimer's disease, where the presence of pathological amyloid and/or tau species in the brain can be detected using positron emission tomography (PET) or cerebrospinal fluid examination and in the near future in blood.^7^ However, these technologies are not quite as reliable as their enthusiasts claim. The proportion of cognitively healthy people aged 80 and over with a positive amyloid biomarker is above 40%.^8^ In a large population-based study, among people with MCI and a positive amyloid PET scan, only one-third progressed to Alzheimer's dementia after nearly 4 years’ follow-up.^8^ In this study, about 15% of those with MCI and a negative amyloid PET scan also progressed to Alzheimer's dementia. Where MCI is applied loosely, i.e. on the basis of symptoms and/or underperformance on a brief cognitive screening instrument rather than comprehensive age-adjusted neuropsychometric tests, the predictive value of biomarkers will be even lower.

Uncertainty over the meaning of an MCI diagnosis is likely to persist despite developments in biomarkers and multi-modal predictive models. Medicalising people by applying a label that signifies potential for the near-term development of an untreatable life-limiting illness that will rob them of cognitive and social abilities risks causing considerable iatrogenic harm.^9^ There is no established intervention to reduce the risk of MCI progressing to dementia, although improved glycaemic and vascular risk factor control in people with diabetes, and some lifestyle and psychosocial interventions, have shown early promise.^10,11^ The skill of the practitioner is thus not just or even particularly in deciding who has MCI; it is in managing the balance of harms and benefits that the label confers and supporting the patient and their supporters to navigate the inevitable uncertainty generated by the diagnosis.

Decisions about whether an MCI label will be a net benefit or harm to a patient should be underpinned by an evidence base on the effects of the diagnosis: what do patients and families recall of discussions at which an MCI diagnosis is disclosed; what does the ‘illness’ of MCI represent to them; and what effects, if any, does have the diagnosis have on their well-being, hopes for the future and sense of agency over their cognitive health?

In the linked article, Munawar and colleagues provide some useful observations from a cohort of nearly 50 patients diagnosed with MCI in a university hospital memory clinic.^12^ Importantly, only one in five MCI patients had instigated the referral process themselves. This reinforces the importance of focusing health promotion about dementia diagnosis on the families and friends of those at risk. The familiar message ‘are you worried about your memory?’ risks iatrogenic harm by lowering the threshold at which people with functional cognitive symptoms develop distress and present to healthcare professionals.

Notably, 60% of patients did not recall the diagnosis of MCI; only 25% saw their symptoms as relating to a pathological process in the brain. Fewer than 20% of patients (and only 25% of accompanying family members) seemed aware that their MCI diagnosis carried a heightened risk of progression to dementia. Only a small minority of patients felt alarmed by the diagnosis, and only one felt stigmatised by the MCI label. Sixty per cent reported no impact on their mood or self-esteem.

These data appear superficially reassuring in that they suggest that the MCI label causes little harm. However, what if patients and families had had better recall of the consultation and a greater understanding of MCI as implying an increased risk of near-term progression to dementia? Perhaps, as Munawar and colleagues suggest, human beings have a natural tendency to construct a narrative in which their condition is ‘controllable’, and this would override attempts to provide information in a more effective way. Nevertheless, the study doesn't rule out the potential for an MCI diagnosis to cause significant harm, especially if accompanied by memorable and persuasive information implying a deterministic relationship with dementia.

Those advocating diagnosing MCI as a means of empowering people to make lifestyle changes to protect their ‘brain health’ will be disappointed by Munawar's findings. Only one in five patients reported taking more exercise or paying greater attention to their health following the diagnosis. If the vast majority of people who have undergone a specialist clinical assessment for dementia and been given an MCI diagnosis don't make any lifestyle adjustments then the likely impact of such advice when given at population level or in primary care will be minimal.

Advocates of so-called ‘brain health services’ propose that providing individuals with a personalised dementia risk assessment followed by lifestyle advice will reduce their risk of progressing to dementia.^13^ In fact, the inevitable effect of a public health strategy based on lifestyle modification in people who happen to attend ‘brain health’ clinics will be to increase health inequalities, as people at the highest risk of illness are also those least likely to use healthcare services and least able to make lifestyle changes. The greatest impact on dementia incidence will come from government action at a population level to reduce poverty, make healthy eating and exercise affordable, limit access to alcohol and tobacco, improve air quality and combat social isolation among other measures. The most important intervention that memory clinics can make is probably to screen patients for hearing loss.^14^ The findings of Munawar and colleagues are mixed in this regard. A significant minority of patients recalled having a hearing screen during the assessment, but the number who were ultimately prescribed and compliant with hearing aids was very small.

In summary, Munawar and colleagues provide cautious optimism that diagnosing more people with MCI will not cause significant iatrogenic harm, although their cohort mostly did not recall their diagnosis or had an overly optimistic understanding of their prognosis. However, their data suggest that the solution to reducing the overall amount of dementia in our communities does not lie in the clinic, as even following a detailed cognitive assessment and explanation, patients are unlikely to take action to reduce their risk of developing dementia.

## About the author

**Jeremy D. Isaacs** is a consultant neurologist at St George's Hospital in London, UK, honorary senior lecturer at St George's, University of London, UK and clinical director of the NHS London dementia clinical network.
